# Active versus passive adverse event reporting after pediatric chiropractic manual therapy: study protocol for a cluster randomized controlled trial

**DOI:** 10.1186/s13063-017-2301-0

**Published:** 2017-12-01

**Authors:** Katherine A. Pohlman, Linda Carroll, Ross T. Tsuyuki, Lisa Hartling, Sunita Vohra

**Affiliations:** 1 Research Institute, Parker University, 2540 Walnut Hill Lane, Dallas, TX 75229 USA; 2 School of Public Health, University of Alberta, 3-300 Edmonton Clinic Health Academy, 11405-87 Ave, Edmonton, AB T6G 1C9 Canada; 3 EPICORE CENTRE, Faculty of Medicine and Dentistry, University of Alberta, 362 Heritage Medical Research Centre, Edmonton, AB T6G 2S2 Canada; 4 Department of Pediatrics, Faculty of Medicine and Dentistry, University of Alberta, 4-472 Edmonton Clinic Health Academy, 11405-87 Ave, Edmonton, AB T6G 1C9 Canada; 5 CARE Program, Department of Pediatrics, Faculty of Medicine and Dentistry, University of Alberta, Suite #1702, College Plaza, 8215 112 St. NW, Edmonton, AB T6G 2C8 Canada

**Keywords:** Pediatrics, Adverse event, Active surveillance, Passive surveillance

## Abstract

**Background:**

Patient safety performance can be assessed with several systems, including passive and active surveillance. Passive surveillance systems provide opportunity for health care personnel to confidentially and voluntarily report incidents, including adverse events, occurring in their work environment. Active surveillance systems systematically monitor patient encounters to seek detailed information about adverse events that occur in work environments; unlike passive surveillance, active surveillance allows for collection of both numerator (number of adverse events) and denominator (number of patients seen) data.

Chiropractic manual therapy is commonly used in both adults and children, yet few studies have been done to evaluate the safety of chiropractic manual therapy for children. In an attempt to evaluate this, this study will compare adverse event reporting in passive versus active surveillance systems after chiropractic manual therapy in the pediatric population.

**Methods/design:**

This cluster randomized controlled trial aims to enroll 70 physicians of chiropractic (unit of randomization) to either passive or active surveillance system to report adverse events that occur after treatment for 60 consecutive pediatric (13 years of age and younger) patient visits (unit of analysis). A modified enrollment process with a two-phase consent procedure will be implemented to maintain provider blinding and minimize dropouts. The first phase of consent is for the provider to confirm their interest in a trial investigating the safety of chiropractic manual therapy. The second phase ensures that they understand the specific requirements for the group to which they were randomized.

Percentages, incidence estimates, and 95% confidence intervals will be used to describe the count of reported adverse events in each group. The primary outcome will be the number and quality of the adverse event reports in the active versus the passive surveillance group. With 80% power and 5% one-sided significance level, the sample size was calculated to be 35 providers in each group, which includes an 11% lost to follow-up of chiropractors and 20% of patient visits.

**Discussion:**

This study will be the first direct comparison of adverse event reporting using passive versus active surveillance. It is also the largest prospective evaluation of adverse events reported after chiropractic manual therapy in children, identified as a major gap in the academic literature.

**Trial registration:**

ClinicalTrials.gov, ID: NCT02268331. Registered on 10 October 2014.

**Electronic supplementary material:**

The online version of this article (doi:10.1186/s13063-017-2301-0) contains supplementary material, which is available to authorized users.

## Background

### Pediatric chiropractic manual therapy and patient safety

Chiropractic manual therapy usually involves the therapeutic application of a force to a pre-determined body structure, which is typically a vertebral or extremity joint. There are numerous manual therapy variations with the velocity, amplitude, loading frequency, choice of lever, location, direction of load, and treatment frequency changing widely amongst the variations [1]. Spinal manipulation therapy (SMT), a type of manual therapy, is regulated for use in many professions (e.g., doctor of osteopathy, medical physicians, and physical therapists), but doctors of chiropractic (DCs) are the most likely to use SMT on a regular basis [2]. According to a 2015 practice analysis of United States DCs, 17.1% of chiropractic patients are 17 years of age or less; this increases to 38.7% amongst DCs who specialize in pediatrics [2, 3].

Adverse events after manual therapy, including SMT, have been investigated more thoroughly in adult patients than in children [4–7]. Several reviews of adverse events in children following manual therapy have identified rare serious adverse events, although the studies have been primarily case reports. The main conclusion from these reviews was that there is insufficient primary research on this topic in this population [8–10].

### Patient safety performance – surveillance systems

To measure safety performance, including reporting of adverse events, many health care settings have implemented surveillance systems to report and learn from adverse events. When established, such systems can provide learning opportunities based on the information gathered [11].

These patient safety surveillance systems vary according to their purpose. *Active surveillance* systematically collects information from the provider about patient encounters, including adverse events, which enhances reporting and demonstrates a health care organization’s commitment to patient safety [11]. Although active surveillance can generate higher quality and quantity of reports because both numerator and denominator data are known, the time and resources needed to properly execute an active surveillance reporting system are often limitations to its successful implementation.

*Passive surveillance* voluntarily collects adverse event information from the provider and is more commonly utilized throughout health care [12]. Typically, passive surveillance systems are conducted confidentially and sometimes anonymously, and some have been modified for Internet-based fora. These systems can also promote quality improvement by allowing for reporting of adverse events, near misses (an event that could have caused an adverse event, but did not), and unsafe conditions. Passive surveillance systems are relatively easy to implement and can collect reports from a broad range of topics and individuals [12]. However, their major limitations include under-reporting (quantity of reports), inadequate information (quality of reports), and limited knowledge of how many patients were exposed (denominator data). Practitioners involved with passive surveillance systems have reported that they commonly forget to write-up their report, are too busy to review others’ reports, are not sure who is responsible to write-up a report, or do not report an event because it seemed trivial [13].

### Study justification

Within the chiropractic profession, active surveillance reporting systems are not used routinely. A passive surveillance system for chiropractic care, called the “Chiropractic Patient Incident Reporting and Learning System” (CPiRLS), is currently being used in Europe and Australia [14, 15]. Although CPiRLS does not have any age restrictions, to date only limited pediatric data have been reported into the system, despite multiple calls for high-quality safety data about pediatric chiropractic manual therapy [8, 10].

Both active and passive surveillance methods have distinct advantages and limitations. The need for a direct comparison of the ability of active versus passive surveillance to report adverse events, and the need to better understand the patient safety performance in the use of chiropractic manual therapies for the pediatric population, led to the development of this cluster randomized clinical trial.

### Study aim and hypothesis

Study aim: to compare the quantity and qualify adverse event reports after chiropractic manual therapy in children 13 years of age or under, using passive versus active surveillance reporting systems. *Hypothesis: DCs randomized to the active surveillance system will report more adverse events and will have better quality reporting than those randomized to the passive surveillance system.*

## Methods

### Study design

The study design is a pragmatic, superiority, cluster randomized clinical trial with a modified enrollment process to maintain participant blinding. DCs in private practice who treat children will be the unit of randomization with random allocation in a 1:1 ratio to active or passive surveillance reporting systems. Cluster randomization was chosen for practical reasons with the unit of analysis being reports from the individual chiropractic patient visits. The University of Alberta’s Research Ethics Board reviewed and approved this study (Pro00027903). The trial has been registered at ClinicalTrials.gov (NCT02268331). The study protocol was prepared using the Standard Protocol Items: Recommendations for Interventional Trials (SPIRIT) guidelines [16] (see Additional file 1) and also the “Methods” section of the Consolidated Standards of Reporting Trials (CONSORT) 2010 Checklist for reporting a cluster randomized controlled trial [17] (see Additional file 2).

### Recruitment, randomization, and enrollment

Licensed DCs in the United States and Canada will be recruited from a variety of venues, including pediatric chiropractic-specific events and organizations, social media, and professional newsletters/magazines. Word of mouth and referrals from colleagues and past participants will also be source of referral into the study.

**Fig. 1:**
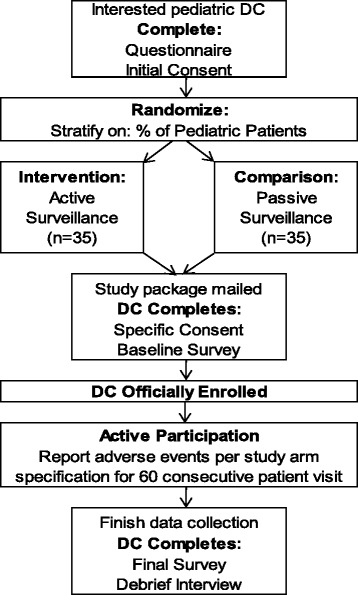
Flow chart of study activities

As shown in Fig. 1, DCs interested in the study will complete a demographic questionnaire and review/sign the initial consent document, which states that they are interested in enrolling in a study to report safety information from 60 consecutive pediatric visits. They will then be randomized to passive or active surveillance by the study coordinator (KAP). To promote baseline equivalence, we will stratify by DC’s self-reported average proportion of pediatric patients seen (>20% versus ≤ 20%). To maintain allocation concealment, the REDCap (Research Electronic Data Capture) Randomization Module will be utilized with a random, variable, permuted block size, generated by an independent biostatistician [18]. Interested DCs will have study materials sent directly to their offices. This material includes the Consent Form that gives details on the surveillance system to which they were randomly assigned. DCs are considered enrolled in the study after that Consent Form is signed and they complete the online baseline survey, which collects additional demographic data and assesses patient safety attitudes [19]. Throughout study participation, to ensure compliance with study methods, regular communications will occur via email or telephone between the study coordinator (KAP) and the DC.

### Intervention arm: active surveillance

For 60 consecutive child patient visits, the parents/caregivers will be given an Information Sheet and asked to complete a pre-treatment form before the child sees the DC. As described in the Information Sheet and as stated on the top of all data collection forms, consent will be implied if the data collection forms are completed and returned. This ensures patient confidentiality. Patients and providers will each be given a post-treatment form to complete. The patient’s post-treatment form is to be completed within 1 week and returned directly to the investigators using a postage-paid envelope. The DC’s post-treatment form is to be completed immediately after the patient’s visit. A more detailed form documenting adverse events will be completed by the provider if a moderate, serious or severe adverse event (see definitions in Table 1) occurs immediately following treatment or is reported to the DC at a later date. All forms (see Additional file 3) were modified from an ongoing, active surveillance study on SMT in Canada [20]. The modified forms were reviewed for content validation by a group of experts, which included the original developers, pediatric chiropractic experts, and caregivers of pediatric chiropractic patients.

**Table 1:** Definitions of terminology for study protocol [20]

Adverse event (AE)	Any unfavorable sign, symptom or disease temporally associated with the treatment, whether or not caused by the treatment. Specifically, any new symptom of moderate severity or a pre-existing symptom that is worse after treatment
Seriousness	*Mild*: asymptomatic or mild symptoms, self-care only (e.g., ice/heat, over-the-counter analgesic)
	*Moderate*: limiting age-appropriate activities of daily living (e.g., work, school); *or* sought care from a physician
	*Severe:* medically significant but not immediately life-threatening; temporarily limits self-care (e.g., bathing, dressing, eating) (for 5 years of age and older); *or* urgent or emergency room assessment sought
	*Serious*: results in death *or* a life-threatening adverse event *or* an adverse event resulting in inpatient hospitalization or prolongation of existing hospitalization for more than 24 h: a persistent or significant incapacity or substantial disruption of the ability to conduct normal life functions; a congenital anomaly/birth defect
Causality (i.e., relatedness)	*Certain*: a clinical event occurring in a plausible time relationship to treatment, and which cannot be explained by concurrent disease or other drugs or therapies
	*Probable/likely:* a clinical event with a reasonable time sequence to treatment, unlikely to be attributed to concurrent disease or other drugs or therapies
	*Possible:* a clinical event with a reasonable time sequence to treatment, but which could also be explained by concurrent disease or other drugs or therapies
	*Unlikely:* a clinical event with a temporal relationship to treatment which makes a causal relationship improbable, and in which drugs, other therapies or underlying disease provide plausible explanations
Preventability	1: Virtually no evidence of preventability
	2: Slight to modest evidence of preventability
	3: Preventability not quite likely (less than 50/50, but “close call”)
	4: Preventability more than likely (more than 50/50, but “close call”)
	5: Strong evidence of preventability
	6: Virtually certain evidence of preventability
Patient disposition	1: Resolved, no sequelae
	2: AE still present – no treatment
	3: AE still present – being treated
	4: Residual effects present – no treatment
	5: Residual effects present – treated
	6: Death
	7: Unknown

### Comparison arm: passive surveillance

The passive surveillance system will use the established *Chiropractic Patient Incident Reporting and Learning System* (CPiRLS) [15]. DCs will be asked to report adverse events that occur in 60 consecutive pediatric patient visits. In this system, only registered providers can submit, read or comment on reports. Participating DCs will be given a universal code to protect anonymity and will also be provided with the CPiRLS’s “trigger list” to advise on what kinds of incidents/adverse events should be reported (see Additional file 4). Reports and comments submitted will be monitored by both the CPiRLS team and the study’s investigators.

### Adjudication

In both the active and the passive groups, when a moderate, severe or serious adverse event is identified, all information from the report will be reviewed independently by blinded content experts to evaluate the event according to the terminology outlined in Table 1 (causality, preventability, and patient disposition). Operational definitions for all terminology were determined through a consensus-based process by the SafetyNET team of manual therapy and patient safety experts [20, 21].

### Outcomes

The primary outcome will be the number (the count) and quality (i.e., ability to meaningfully interpret/adjudicate, a binary variable) of the DC’s adverse event(s) reports per patient visit and per patient in each group. Quality of adverse event reports will be assessed by the adjudicators’ ability to meaningfully adjudicate the report (section above).

A secondary outcome is the change in patient safety attitudes for participating DCs. This will be measured in both groups using the Safety Organizing Scale [19], which is a nine-item survey with a 7-point rating scale (1 – “Not at all”; 7 – “To a very great extent”). This questionnaire is to be completed at two time points: at baseline (the online baseline survey prior to study enrollment) and after adverse event data collection is complete for each participating DC. In the active surveillance arm, additional variables to assess adverse events and associated factors for adverse events include: patient-reported adverse events, manual therapy treatment description, patient health history, and patient satisfaction [22].

### Minimization of systematic error

To reduce potential respondent bias and maximize data integrity, a modified enrollment process will be utilized with a two-phased consent process. The first phase has a consent document focused on safety outcomes data collection rather than a comparison of the two different methodologies for collecting such outcomes. This focus is utilized to both blind participants to the comparison under evaluation and minimize dropouts as one arm (active surveillance) is more time intensive than the other (passive surveillance), but both arms are enhancements to current standard of North America practices. The second phase occurs after randomization with the consent document explaining the exact study procedures of the participant’s allocated group without reference to the other group. There will be a debrief interview at the end of a DC’s study participation to explain this modified enrollment process and the procedure for both study groups.

Other study personnel who will be blinded in the study include: (1) patients, (2) an independent biostatistician for analysis, and (3) content experts involved in the adjudication process. Because of the major differences in data management, the investigator (KAP) responsible for study coordination cannot be blinded.

### Clinical data management

All data will be entered and managed using REDCap electronic data capture tools, which is hosted at the University of Alberta [18]. REDCap is a secure, web-based application designed to support data capture for research studies.

For the active surveillance group, the data will be verified and validated, and the quality checked by a single study investigator (KAP) who will compare the patient’s pre- and post-treatment forms to ensure that inconsistencies are corrected. For audit purposes and to ensure transparency, all changes made will be recorded with the time and date and user ID. The study investigator will discuss any queries with the study team with query resolutions recorded.

### Statistical methods

The count of reported adverse events (any severity) in each group will be expressed with percentages and incidence estimates, and their 95% confidence intervals (CIs). The primary analysis will compare the cumulative incidence of adverse event reports in active versus passive surveillance. Because the outcome is number of events, it is assumed that the data will follow a Poisson distribution. Hence, a Poisson regression with log links will be used in general estimating equation (GEE) analyses with an appropriate sandwich estimator to take into account the DC cluster correlation. Groups will be compared using an intention-to-treat analysis.

Sensitivity analysis, using the same GEE analyses as above, will be conducted for reports that were not adjudicated (because of uninterpretable adverse events) and differences in how missing data were handled (i.e., imputing using average incidence and highest incidence). The binary variable expressing if the quality of the adverse event report allowed for meaningful interpretation/adjudication will be evaluated using the McNemar’s exact test because of the expected rarity of reports and cluster correlation.

Secondary analysis will address differences in the count of adverse event reports by patient-only, provider-only, and those reported by the active surveillance versus the provider-reports in the passive surveillance. Like the primary analyses, Poisson regression with log links will be used in GEE analyses to account for cluster-specific methods. Patient safety attitudes will be measured before and after participation and compared across surveillance groups.

Other planned secondary analyses are designed to identify factors associated with adverse events from the data gathered in the active surveillance group. Potential factors for adverse events include patient characteristics (e.g., age, presenting condition, sex, health history), provider characteristics (e.g., years in practice, specialty training), and treatment provided (e.g., high-velocity, low-amplitude or other). With the adverse event reports categorized by their severity (i.e., none, mild, moderate, severe, serious), logistic regression analyses will be used to model factors associated with the adverse events. If the number of moderate, severe, and serious events are small, the outcome will be dichotomized as any adverse event versus no adverse event. If numbers of moderate, severe and serious events are sufficiently large, multivariable polytomous logistic regression will be used.

Planned exploratory analyses include: (1) subgroup analysis for providers with a specialty pediatric certification and number of reported adverse events (i.e., the primary outcome); (2) assessment of the feasibility to implement a surveillance system within chiropractic offices from individual provider feedback; and (3) review of debrief interview to gain insight into participating DCs’ overall thoughts on the study, including barriers to implementation, perceived benefit of participating, and being blinded to intervention. An assessment of bias will be conducted with responding and non-responding patient demographic characteristics for the active surveillance group. All analyses will be conducted using Stata version 13 (StataCorp LP, College Station, TX, USA).

### Sample size

An estimated active surveillance reporting rate of 4.3% and intracluster correlation of *ρ* = 0.13 were based on a pilot study of a similar active surveillance used within the chiropractic profession in Canada [20, 21]. We assumed a passive surveillance reporting rate of 0.53%, based on prior academic literature [8]. A one-sided significance level was utilized as it seems reasonable to believe that passive surveillance will result in under-reporting of adverse events [23]. We calculated that a sample size of 35 providers in each group, with each DC collecting data from 60 pediatric patient visits, and 5% one-sided significance level, would lead to 80% power. This includes an anticipated loss to follow-up of 11% of DCs and 20% of patient visits.

## Discussion

This study will be the largest prospective evaluation of adverse events reported after chiropractic manual therapy in the pediatric population, which has been identified as a major gap in the academic literature [8–10, 24]. This randomized cluster trial assesses the effectiveness of two different surveillance methods to collect observational safety data on a topic that is clinically relevant. To our knowledge, this is the first study to do a direct comparison of active versus passive surveillance reporting of adverse events.

The chiropractic profession treats children [3, 25]; therefore, it has a responsibility to ensure proper safety evaluations. The attitudes and opinions of DCs, who are interested in pediatric treatment, for implementing safety performance systems were evaluated in 2014. The survey identified a robust patient safety climate with time pressure as the barrier of most concern to participants [26]. Time pressure is a common barrier for health care provider participation in research, as “busy-ness” is seen as a socially acceptable excuse for declining “extra” activities [27]. Our study protocol took this concern into consideration. When pilot tested, passive surveillance was found to add 30 s per patient visit while active surveillance added only 2 min [20].

Aside from reports of actual adverse events that are collected in this study, each surveillance method also collects additional patient safety information. While not the primary outcome, this study will also clearly describe and report these differences. Such examples from the passive surveillance group includes administrative, incidental patient safety incidents (e.g., use of the wrong clinical file or tripping over office equipment) or “near misses”/events, which could have caused an adverse event, but did not. For the active surveillance group, information will be sought not only from the DC, but also directly from the patients; patient-provided information can be compared to that information known by the provider. These differences are unique to each surveillance group and should be taken in consideration when an organization is deciding on what method to use to evaluate adverse event.

Beyond the significance of the study’s specific aims, the study procedures also include several notable methodological considerations, such as the attention to outcome measurement and a modified enrollment process to maintain participant blinding. This study started with a content validation of the data collection instruments to ensure that they will collect the intended information and that it will be easily understood by the chiropractic pediatric patient’s parent/caregiver [28].

Modified enrollment procedures have been utilized most commonly to avoid biases that occur with non-placebo-controlled trials [29]. This study will use a modified enrollment procedure, a two-stage consent process, to ensure that provider blinding is maintained and dropouts minimized. To avoid ethical concerns regarding enrolling and randomizing providers without their consent, consent is sought in two stages: first, providers consent to participation in a study on pediatric patient safety and chiropractic manual therapy. The second consent will give full disclosure of their specific study procedures. When participant’s complete the study, a debrief interview will unveil the two groups and the purpose for not disclosing this information earlier.

### Barriers to study completion

Possible barriers to the study’s implementation will be the willingness of DCs to participate in research and their adherence to study procedures. Adherence will be addressed by actively following up on DCs interested in this study’s topic, engaging front desk personnel in study processes, and assuring that the study protocol is understood. Despite these precautions, compliance is expected to be challenging, specifically for chiropractic practices that are assigned to the active surveillance group. Dropouts have been taken into account in the sample size calculations.

Another concern regarding the study’s implementation is the possibility of a low response rate for the active surveillance arm’s post-treatment form, to be completed by the patient’s caregiver. The pilot study found that DCs who encouraged their patients to complete the data collection instruments had a better response rate [20, 21].

## Trial status

This is the first version of the study protocol. Modification or amendments that have an impact on the conduct of the study will be documented and described in further publications. At the time of protocol submission, this trial was in active recruitment.

## Additional files

Additional file 1: SPIRIT 2013 Checklist. (PDF 48 kb)

Additional file 2: CONSORT 2010 Checklist. (PDF 137 kb)

Additional file 3: Active Surveillance Study Forms. (PDF 23072 kb)

Additional file 4: Passive Surveillance Trigger List. (PDF 365 kb)

## Abbreviations

CPiRLS: Chiropractic Patient Incident Reporting and Learning System
DC: Doctor of chiropractic
GEE: General estimating equation
REDCap: Research Electronic Data Capture
SMT: Spinal manipulation therapy
