# Supplementary material for: Active versus passive adverse event reporting after pediatric chiropractic manual therapy: study protocol for a cluster randomized controlled trial
Source: Trials. 2017 Dec 1;18:575. doi: 10.1186/s13063-017-2301-0 (PMC5709959; doi:10.1186/s13063-017-2301-0)
Supplement: Supplementary file 3 — Active Surveillance Study Forms. (PDF 23072 kb) [file 13063_2017_2301_MOESM3_ESM.pdf]

SafetyNET Patient Information Sheet - Pediatric

Title of Study: Improving the Assessment of Safety in Pediatric Chiropractic Manual Therapy

Investigators:
Dr. Katherine Pohlman (780-342-8447)
Dr. Sunita Vohra (780-342-8592)
Dr. Linda Carroll (780-492-9767)

Why is your child being asked to take part in this research study?

Your child is being asked to take part in this research study being done with the University of Alberta because your chiropractor is a research participant in this study. The purpose of the study is to collect information related to children patient's experience of chiropractic manual therapy, including safety information. Your participation is voluntary and completion / return of the form means you agree to be part of this study.

What is the reason for doing the study?

The study is being done to support patient safety which will allow doctors of chiropractic to identify concerns and find ways to reduce them.

What will I be asked to do?

If you agree to take part on behalf of your child, we will ask you to complete 2 forms about your child's visit with your chiropractor today. The forms will only take a few minutes to fill out. The PRE form will be completed before your child sees the chiropractor. The POST form will ask you to comment on any effects / problems / symptoms your child may have experienced during or after his / her treatment. This form can be completed and returned at any time, using the pre-addressed and postage paid envelope; however, we are most interested in your feedback one week after your child's visit, but prior to your child's next visit with this chiropractor.

What are the risks and discomforts?

There are no known risks associated with participating in this study.

What are the benefits to me?
You and your child are not expected to get any benefit from being in this research study. The benefits of taking part are to help increase our knowledge about the potential effects and safety of chiropractic manual therapy.

Do I have to take part in the study?
Being in this study is your choice. If you decide your child should be in the study, you can change your mind and stop being in the study at any time, and it will not affect you or your child's treatment. No one, including the researcher, will know whether or not you have participated.

Will my information be kept private?
During the study, if you choose to complete the forms on behalf of your child, we will collect data about the care he or she received from your chiropractor. We will do everything we can to make sure that this data is kept private. No data relating to this study includes your child's name. Sometimes, by law, we may have to release our data and the information you provided, so we cannot guarantee absolute privacy. However, we will make every legal effort to make sure that your child's information is kept private. Furthermore, your chiropractor identified by a code, rather than by name, and this identifying code will be destroyed 90 days after their participation in the study. After that time, we will not have any record of who took part in the study. Thus, we will not be able to connect your chiropractor to any of your child's visit after this time.

What if I have questions?
If you have concerns about this research now or later, please contact the study coordinator, Dr. Katie Pohlman at 780-342-8447. If you have any questions regarding your rights as a research participant, you may contact the Health Research Ethics Board at 780-492-2615. This office has no affiliation with the study investigators.

Thank you for your support.
Your feedback is extremely valuable.

Completing this form means you agree to be part of this study. Please give to your chiropractor before your visit starts.

1. This form is being completed for this child by: ◯ Mother ◯ Father ◯ Other, specify: ____________________

2. Please mark the reason(s) for your child's appointment today:
   ◯ Preventative / Wellness / No Symptoms ◯ Headache ◯ Neck pain ◯ Mid-back pain
   ◯ Low-back pain ◯ Arm / Shoulder / Knee / Leg Pain ◯ ADD / ADHD ◯ Autism
   ◯ Breastfeeding Difficulties ◯ Cold ◯ Colic ◯ Digestive Issues ◯ Plagiocephaly
   ◯ Torticollis ◯ Other, specify: ____________________

3. How long has your child had this condition? __________ week(s) ◯ >1 year ◯ N/A

4. How many treatments has your child had for this condition? __________ treatments ◯ N/A
   Over what period of time? __________ week(s) ◯ >1 year ◯ N/A

5. In the past 7 days, how would you rate your child's pain on average?

0	1	2	3	4	5	6	7	8	9	10
☐	☐	☐	☐	☐	☐	☐	☐	☐	☐	☐
No pain										Worst imaginable pain

6. Please indicate any medications that your child is taking: ◯ None
   ◯ Acetaminophen / Ibuprofen ◯ Cetirizine (Zyrtec/Reactine) ◯ Diflucan ◯ Gaviscon
   ◯ Omeprazole (Prilosec, Losec) ◯ Ranitidine (Zantac) ◯ Other: ____________________

7. Please indicate any vitamins or natural health products that your child is taking: ◯ None
   ◯ Omega-3 ◯ Probiotics ◯ Vitamin D ◯ Other: ____________________

8. Does your child have a history of any of the following? ◯ None
   ◯ Bleeding disorder ◯ Cancer ◯ Diabetes ◯ Other: ____________________

9. Child is: ◯ Male ◯ Female ◯ Other: ____________________

10. Child's date of birth? ____Month ____Day 20____ ____

11. Today's fees covered by: ◯ N/A ◯ Self-pay ◯ Car Accident Coverage ◯ Other Insurance: ____________________

Please continue with questions on the back.

**First:** Does your child have any of the following? (check all that apply)	**Second:** For each item checked, please answer the questions below		
	How long has your child had it?	Does it interfere with their usual daily activities (e.g. play, school)?	Does it limit their ability to care for themselves (e.g. bathing, dressing, eating)?
◯ Discomfort / Pain	________ week(s) ◯ >1 year	◯ Yes ◯ No	◯ Yes ◯ No
◯ Stiffness	________ week(s) ◯ >1 year	◯ Yes ◯ No	◯ Yes ◯ No
◯ Weakness	________ week(s) ◯ >1 year	◯ Yes ◯ No	◯ Yes ◯ No
◯ Fatigue / Tiredness	________ week(s) ◯ >1 year	◯ Yes ◯ No	◯ Yes ◯ No
◯ Headache	________ week(s) ◯ >1 year	◯ Yes ◯ No	◯ Yes ◯ No
◯ Dizziness	________ week(s) ◯ >1 year	◯ Yes ◯ No	◯ Yes ◯ No
◯ Numbness / Tingling	________ week(s) ◯ >1 year	◯ Yes ◯ No	◯ Yes ◯ No
◯ Problems Sleeping	________ week(s) ◯ >1 year	◯ Yes ◯ No	◯ Yes ◯ No
◯ Irritability / Crying	________ week(s) ◯ >1 year	◯ Yes ◯ No	◯ Yes ◯ No
◯ Other: ________ ________	________ week(s) ◯ >1 year	◯ Yes ◯ No	◯ Yes ◯ No
◯ None of the above			

STOP

Please give the form to the CHIROPRACTOR, who will complete the remainder *below*:

① **Radicular Pain?** ◯ Yes ◯ No ② **Please indicate if the main condition is:** ◯ Chronic ◯ Acute ◯ Recurring ◯ NA

**TREATMENT**	**Cervical Spine**	**Thoracic Spine**	**Lumbar Spine**	**Sacrum / Pelvis**	**Upper Extremity**	**Lower Extremity**	**Other ***
**# of Manipulations**	◯1 ◯2 ◯3+	◯1 ◯2 ◯3+	◯1 ◯2 ◯3+	◯1 ◯2 ◯3+	◯1 ◯2 ◯3+	◯1 ◯2 ◯3+	◯1 ◯2 ◯3+
**# of Mobilizations**	◯1 ◯2 ◯3+	◯1 ◯2 ◯3+	◯1 ◯2 ◯3+	◯1 ◯2 ◯3+	◯1 ◯2 ◯3+	◯1 ◯2 ◯3+	◯1 ◯2 ◯3+
**Mechanical Device**	◯1 ◯2 ◯3+	◯1 ◯2 ◯3+	◯1 ◯2 ◯3+	◯1 ◯2 ◯3+	◯1 ◯2 ◯3+	◯1 ◯2 ◯3+	◯1 ◯2 ◯3+
**Other Manual Tx***	◯1 ◯2 ◯3+	◯1 ◯2 ◯3+	◯1 ◯2 ◯3+	◯1 ◯2 ◯3+	◯1 ◯2 ◯3+	◯1 ◯2 ◯3+	◯1 ◯2 ◯3+
**Other Non-Man. Tx***	◯1 ◯2 ◯3+	◯1 ◯2 ◯3+	◯1 ◯2 ◯3+	◯1 ◯2 ◯3+	◯1 ◯2 ◯3+	◯1 ◯2 ◯3+	◯1 ◯2 ◯3+

***Other**, (specify) ________________________________

POST SYMPTOMS

Was there any adverse event after the manual therapy treatment? ◯ No ◯ Yes (complete table below)

**Post Symptoms** (check all that apply)	**Pre-Existing:**	**If Yes: Better, Worse, or Unchanged?**	**Anticipated?**	**Overall Severity Rating**
◯ Discomfort / Pain	◯ Yes ◯ No	◯B ◯W ◯U	◯ Yes ◯ No	◯ Mild ◯ Moderate ◯ Severe ◯ Serious
◯ Stiffness	◯ Yes ◯ No	◯B ◯W ◯U	◯ Yes ◯ No	◯ Mild ◯ Moderate ◯ Severe ◯ Serious
◯ Weakness	◯ Yes ◯ No	◯B ◯W ◯U	◯ Yes ◯ No	◯ Mild ◯ Moderate ◯ Severe ◯ Serious
◯ Fatigue / Tiredness	◯ Yes ◯ No	◯B ◯W ◯U	◯ Yes ◯ No	◯ Mild ◯ Moderate ◯ Severe ◯ Serious
◯ Headache	◯ Yes ◯ No	◯B ◯W ◯U	◯ Yes ◯ No	◯ Mild ◯ Moderate ◯ Severe ◯ Serious
◯ Dizziness	◯ Yes ◯ No	◯B ◯W ◯U	◯ Yes ◯ No	◯ Mild ◯ Moderate ◯ Severe ◯ Serious
◯ Numbness / Tingling	◯ Yes ◯ No	◯B ◯W ◯U	◯ Yes ◯ No	◯ Mild ◯ Moderate ◯ Severe ◯ Serious
◯ Irritability / Crying	◯ Yes ◯ No	◯B ◯W ◯U	◯ Yes ◯ No	◯ Mild ◯ Moderate ◯ Severe ◯ Serious
◯ Other: ________ ________	◯ Yes ◯ No	◯B ◯W ◯U	◯ Yes ◯ No	◯ Mild ◯ Moderate ◯ Severe ◯ Serious

Please put completed form in SafetyNET Box.

Thank you for your support.
Your feedback is extremely valuable.

- Completion and return of this form means you agree to be part of this study on behalf of this child. Please answer the questions based upon the appointment date identified below.
- You can complete this survey at any time; however, we are most interested in the feedback **one week after your child's visit, but before his / her next visit**.

1. Date of completion: _____ _____ / _____ _____ / 201_____ (Month / Day / Year)

2. This form is being completed for this child by: ◯ Mother ◯ Father ◯ Other: ______________________

Mark only one checkbox on each line	Very satisfied	Somewhat satisfied	Neither satisfied nor dissatisfied	Somewhat dissatisfied	Very dissatisfied
3. How satisfied are you with the information you have been given from your child's chiropractor?	◯	◯	◯	◯	◯
4. How satisfied are you with the treatment(s) that your child received?	◯	◯	◯	◯	◯
5. How satisfied are you with the overall care that your child received?	◯	◯	◯	◯	◯

6. In the past 7 days, how would you rate your child's pain on average?

0	1	2	3	4	5	6	7	8	9	10
☐	☐	☐	☐	☐	☐	☐	☐	☐	☐	☐
No pain										Worst imaginable pain

7. During the appointment with the chiropractor, did your child receive **manual therapy** (also called manipulation, mobilization or adjustment; defined as '*A hands-on therapy to affect joints in the neck, back or limbs; sometimes hand-held mechanical devices are also used.*')?:

◯ **No**

◯ **Yes**, please mark all areas where you received a manual therapy:

◯ Neck ◯ Back ◯ Shoulder/Arms/Knee/Legs ◯ Other: ______________________

8. Since your child's appointment with the chiropractor, what other treatments / therapies has your child had?

◯ None

◯ Other **manual therapy** (also called manipulation, mobilization or adjustment; defined as '*A hands-on therapy to affect joints in the neck, back or limbs; sometimes hand-held mechanical devices are also used.*')
please specify: ______________________

◯ New medicine, please specify: ______________________

◯ New natural health products, please specify: ______________________

◯ Other, please specify: ______________________

9. How would you describe the overall effect of your child's visit with your chiropractor?

◯ Favorable ◯ Unfavorable ◯ None ◯ Unsure

Please complete page 2 (on reverse)

① **FIRST:** Sincethe appointment did your child have any of the following? (check all that apply below)	② Did you expect this to occur in your child?	③ If symptom was there before treatment, is it now: **B**etter, or **W**orse, **U**nchanged or **N**ot there before treatment?	④ Did it interfere with your child's usual daily activities (e.g. work, school)?	⑤ Did it limit your child's ability to care for them self (e.g. bathing, dressing, eating)?	⑥ Did your child need to see a medical doctor because of it?	⑦ Was your child admitted to hospital because of it?	⑧ How many hours after the therapy did it start?	⑨ How long did it last? (check 'C' if it still continues)		If 'C', is your child currently being treated for it?
	**SECOND:** For each item you checked 'yes' in the first column, please answer the questions *below*.									
**Discomfort/Pain** Yes / No	Yes / No	B W U N	Yes / No	Yes / No	Yes / No	Yes / No	___ hour(s)	___ hour(s)	C	Yes / No
**Stiffness** Yes / No	Yes / No	B W U N	Yes / No	Yes / No	Yes / No	Yes / No	___ hour(s)	___ hour(s)	C	Yes / No
**Weakness** Yes / No	Yes / No	B W U N	Yes / No	Yes / No	Yes / No	Yes / No	___ hour(s)	___ hour(s)	C	Yes / No
**Tiredness/Fatigue** Yes / No	Yes / No	B W U N	Yes / No	Yes / No	Yes / No	Yes / No	___ hour(s)	___ hour(s)	C	Yes / No
**Headache** Yes / No	Yes / No	B W U N	Yes / No	Yes / No	Yes / No	Yes / No	___ hour(s)	___ hour(s)	C	Yes / No
**Dizziness** Yes / No	Yes / No	B W U N	Yes / No	Yes / No	Yes / No	Yes / No	___ hour(s)	___ hour(s)	C	Yes / No
**Numbness/Tingling** Yes / No	Yes / No	B W U N	Yes / No	Yes / No	Yes / No	Yes / No	___ hour(s)	___ hour(s)	C	Yes / No
**Problems Sleeping** Yes / No	Yes / No	B W U N	Yes / No	Yes / No	Yes / No	Yes / No	___ hour(s)	___ hour(s)	C	Yes / No
**Irritability/Crying** Yes / No	Yes / No	B W U N	Yes / No	Yes / No	Yes / No	Yes / No	___ hour(s)	___ hour(s)	C	Yes / No
**Other Condition** ______________ No	Yes / No	B W U N	Yes / No	Yes / No	Yes / No	Yes / No	___ hour(s)	___ hour(s)	C	Yes / No

Thank you for participating in the SafetyNET study. Please seal in envelope provided and place in mail.

SafetyNET PEDIATRICS

Complete ONLY for Moderate, Severe or Serious Adverse Events

- Please fax completed forms to: **214-902-2482**
- Completion of this form does not replace your usual communication with your insurance group.

GENERAL ADVERSE EVENT NARRATIVE

(1) Please describe what happened. (Include date of onset, manual therapy technique / location, treatment schedule, patient's response, tests done to evaluate the symptoms, and all actions taken.)

(2) How long after treatment did the adverse event occur?: ____________________ Hours OR ____________________ Days

(3) In your opinion, what may have contributed to the adverse event?

____

____

____

PATIENT CHARACTERISTICS Please describe what was known *PRIOR TO* treatment

(4) Reason of patient visit: ____

(5) What was patient's specific diagnosis for treatment? (Include details such as acute / chronic / recurring, what symptoms they had, and what diagnostic tests were done prior to treatment.)

____

____

____

____

(6) Has the patient experienced an adverse event to manual therapy in the past?

○ Yes ○ No ○ Unknown ○ **If yes**, please specify ________________________________

Please continue with questions on the back.

PATIENT CHARACTERISTICS con't – Please describe what was known *PRIOR TO* treatment

(7) Did the patient have any other diagnoses?

○ Yes ○ No ○ Unknown ○ **If yes**, please specify: ______________________________

(8) Were you aware if the patient had any of the following conditions **prior to treatment**?

○ Acute infection
○ Bleeding tendency
○ Connective tissue disorder
○ Diabetes
○ Fracture
○ History of cancer
○ History of stroke
○ Prior spine surgeries
○ Radiculopathy
○ Recent relevant trauma
○ Recent upper respiratory infection
○ Vertigo
○ Fever
○ Other ______________________

(9) Please check medication(s) or natural health product(s) the patient was taking **prior to treatment**.

Prescription Medications

○ Don't Know
○ Acetaminophen / Ibuprofen
○ Ceririzine (Zyrtec)
○ Diflucan
○ Gaviscon
○ Omerprzole (Prolosec, Losec)
○ Ranitidine (Zantac)
○ Other, Specify ______________

Natural Health Products

○ Don't Know
○ Omega 3 Fatty Acids
○ Probiotics
○ Vitamin D
○ Other NHP, Specify:
____
____

OUTCOME (from your perspective / awareness)

PATIENT IMPACT:

(10) What activities of daily living were affected? ______________________________

(11) Was self-care affected? ○ Yes ○ No ○ Unknown

(12) Was the patient hospitalized? ○ Yes ○ No ○ Unknown

(13) Describe any residual effect / permanent disability / death: ______________________________

(14) Did the adverse event require treatment? ○ Yes ○ No ○ Unknown

(15) Has the adverse event resolved? ○ Yes ○ No ○ Unknown

If Yes, Date of Resolution (dd/mm/yyyy) ____ ____ / ____ ____ / 201 ____

PROVIDER IMPACT:

(16) Has this event caused you to make any changes to your practice? ○ Yes ○ No

If Yes, describe: ______________________________

(17) Were there factors that could have minimized / prevented this event? ○ Yes ○ No

If Yes, describe: ______________________________
