# Supplementary material for: Active versus passive adverse event reporting after pediatric chiropractic manual therapy: study protocol for a cluster randomized controlled trial
Source: Trials. 2017 Dec 1;18:575. doi: 10.1186/s13063-017-2301-0 (PMC5709959; doi:10.1186/s13063-017-2301-0)
Supplement: Supplementary file 4 — Passive Surveillance Trigger List. (PDF 365 kb) [file 13063_2017_2301_MOESM4_ESM.pdf]

www.cpirls.org

Incidents that have occurred

Incidents that almost occurred (near miss)

Incidents that could occur

CPiRLS Trigger List

The trigger list below is designed to help you participate in CPiRLS by providing examples of incidents that you might experience and should report. Some incidents may be fairly common while some may be extremely rare. The list is not exhaustive but provides the categories/subcategories of incident that match those examples listed in the online reporting form. These same categories/subcategories can be applied to near misses and potential incidents on the understanding that you are referring to the *avoidance* of the incident or an *identified risk* of a particular incident occurring.

DOCUMENTATION	EXAMINATION/ ASSESSMENT	TREATMENT/ MANAGEMENT	ACCIDENTS/ EQUIPMENT/ INFRASTRUCTURE
Patient record inadequate	Incorrect diagnosis	Patient experienced post-treatment distress/pain	Patient trip/fall
Failure to take notes on a new episode	Investigation undertaken to detriment of patient	Wrong positioning of patient during treatment	Patient unable to contact clinic in an emergency
Failure to document diagnosis / prognosis	Significant pathology missed	Patient experienced significant post treatment effects e.g. neurological problem, disc prolapsed	Equipment malfunction
Patient record misplaced	Case history inadequate, missed secondary condition	Patient experienced negative effects during treatment e.g. fractured rib or clavicle	Failure to use equipment appropriately
Records confused, treated wrong patient	Over-exposure of film	Suggested drugs to patient which had adverse effect	Health and Safety measures inadequate
Treated before referral notes arrived, missed significant finding	Over-exposure of patient	Did not modify treatment plan to take account of patient preferences or health needs	Failure to dispose of sharps and clinical waste appropriately
Failure to gain consent	Failure to request x-ray report	Slow to refer after patient did not respond to treatment	Medical emergency inadequately handled
Breach of confidentiality	X-ray misinterpretation	Did not discontinue treatment when appropriate to do so	Exposure to blood
	Exposure of pregnant patient	Patient discharged without arranging future care	Exposure to harmful substances
	Failure in referral process		
